# County-level hurricane exposure and birth rates: application of difference-in-differences analysis for confounding control

**DOI:** 10.1186/s12982-015-0042-7

**Published:** 2015-12-22

**Authors:** Shannon C. Grabich, Whitney R. Robinson, Stephanie M. Engel, Charles E. Konrad, David B. Richardson, Jennifer A. Horney

**Affiliations:** Department of Epidemiology, Gillings School of Global Public Health, University of North Carolina at Chapel Hill, 135 Dauer Drive 2101 McGavran-Greenberg Hall, CB #7435, Chapel Hill, NC 27599 USA; Department of Geography, University of North Carolina at Chapel Hill, Chapel Hill, NC USA; Department of Epidemiology and Biostatistics, School of Rural Public Health, Texas A&M Health Science Center, College Station, TX USA

**Keywords:** Difference-in-difference, Fixed-effect regression, General linear models, Hurricane, Disaster, Birth rates

## Abstract

**Background:**

Epidemiological analyses of aggregated data are often used to evaluate theoretical health effects of natural disasters. Such analyses are susceptible to confounding by unmeasured differences between the exposed and unexposed populations. To demonstrate the difference-in-difference method our population included all recorded Florida live births that reached 20 weeks gestation and conceived after the first hurricane of 2004 or in 2003 (when no hurricanes made landfall). Hurricane exposure was categorized using ≥74 mile per hour hurricane wind speed as well as a 60 km spatial buffer based on weather data from the National Oceanic and Atmospheric Administration. The effect of exposure was quantified as live birth rate differences and 95 % confidence intervals [RD (95 % CI)]. To illustrate sensitivity of the results, the difference-in-differences estimates were compared to general linear models adjusted for census-level covariates. This analysis demonstrates difference-in-differences as a method to control for time-invariant confounders investigating hurricane exposure on live birth rates.

**Results:**

Difference-in-differences analysis yielded consistently null associations across exposure metrics and hurricanes for the post hurricane rate difference between exposed and unexposed areas (e.g., Hurricane Ivan for 60 km spatial buffer [−0.02 births/1000 individuals (−0.51, 0.47)]. In contrast, general linear models suggested a positive association between hurricane exposure and birth rate [Hurricane Ivan for 60 km spatial buffer (2.80 births/1000 individuals (1.94, 3.67)] but not all models.

**Conclusions:**

Ecological studies of associations between environmental exposures and health are susceptible to confounding due to unmeasured population attributes. Here we demonstrate an accessible method of control for time-invariant confounders for future research.

**Electronic supplementary material:**

The online version of this article (doi:10.1186/s12982-015-0042-7) contains supplementary material, which is available to authorized users.

## Background

Ecological analyses with exposure and outcome measures at aggregate level are often used in environmental and natural disaster epidemiology. The purpose of aggregate level analyses can be the estimation of ecological associations or inference to individual risks. While aggregate level research may be more practical when individual exposures and outcomes are difficult to define, there are many methodological challenges surrounding its use in inference on individual risks. Some concerns may include ecological bias, exposure misclassification and proper control of measured or unmeasured confounders. Challenges inherent to the timely collection of post-disaster data or reliance on surveillance data often leads to lack of control for unmeasured confounding.

To assess the health impacts of hurricanes and inform the policies needed to mitigate adverse effects, epidemiologists often conduct analyses of aggregated data [1, 2]. The findings of the current literature on hurricane exposure and reproductive health outcomes are generally mixed [3–7]. These inconsistencies may be in part the result of the limitations associated with the use of aggregate data such as determination of timing in exposure and outcome relationships. Methods like difference-in-differences fixed-effects modeling can be applied to control for unmeasured confounding in pre-post or county-level level analysis [8–10].

Difference-in-differences methods have a long history in disciplines outside of epidemiology [11–13]; however, their use is relatively less common in epidemiology, with the exception of a few studies [14–16] as well as case-crossover and case-time-control designs. Difference-in-differences methods can be applied to any model where outcomes are observed in a minimum of two groups (e.g., treatments or exposure categories) at two different time points assuming confounders are time invariant [17–19].The exposed group must have an exposure status which changes across the two time points, while the referent group remains unexposed in both time periods. The estimate in the unexposed group is then subtracted from that of the exposed group. This removes biases resulting from static population characteristics between the two time points. A commentary by Kaufman discusses the application of similar fixed-effects methods in epidemiology to reduce bias and derive more valid estimates [20]. This method is a relatively simple yet powerful technique to address confounding inherent in comparing populations that may not have the same baseline characteristics.

To demonstrate an application of this method in county-level analysis, we assessed the association between hurricane exposure and live birth rates. Live birth rates are often anecdotally assumed to be influenced by natural disaster occurrences, with some reports suggesting a “baby boom” following severe weather events [5, 21]. In other words, live birth rates may increase after disaster occurrence through increased conception rates. We compared an adjusted general linear model approach, to directly compare birth rates in counties affected and unaffected by hurricanes, to results obtained by a difference-in-differences analysis to illustrate the method’s application for future epidemiology research.

## Methods

### Study population

We used a retrospective cohort of 2003 and 2004 Florida conceptions resulting in live birth to demonstrate the difference-in-differences method on the relationship between county-level hurricane exposure and live birth rates. Four hurricanes made landfall in Florida during the 2004 hurricane season, exposing the majority of the 67 counties to hurricane weather. No hurricanes made landfall during the 2003 season. Therefore conceptions during 2003 were all considered unexposed while conceptions during 2004 could be considered exposed or unexposed depending on maternal county of residence. Our source population, from vital records data, included all documented Florida pregnancies conceived in 2003 and 2004 that completed a minimum of 20 weeks gestation.

The 2004 cohort used in both the difference-in-differences models and general linear models included women who conceived between August 14, 2004 and October 31, 2004. Conception was estimated based on clinical estimate from the birth certificate. The defined window of exposure falls from just after the first hurricane occurrence through 3 months after the last hurricane occurrence. This exposure window aligned with the conception-based “baby-boom” hypothesis. For the difference-in-differences analysis, we also used pregnancies conceived in the previous year, from August 14, 2003 and October 31, 2003, to calculate 2003 unexposed live birth rates.

### Exclusions

We excluded births to non-Florida residents, as they did not have a residential address to link to Florida hurricane exposure. Additionally, births with gestational age less than 20 weeks and to mothers less than 15 years at delivery or greater than 45 years of age were also excluded. Of the 94,593 total eligible births, 92,398 remained in the analytic population after exclusion criteria were applied.

### Hurricane exposure

**Fig. 1:**
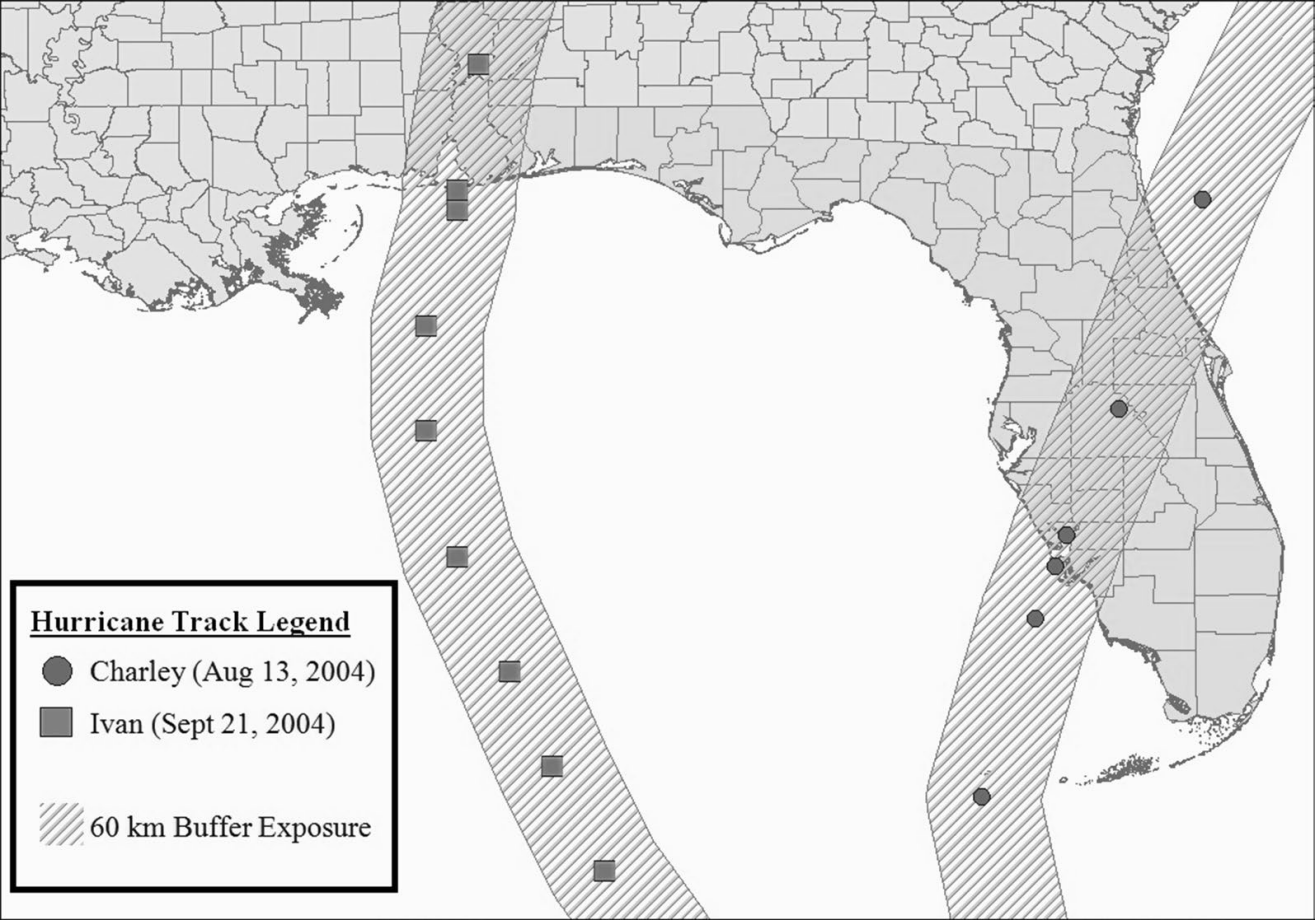
2004 Florida track map with 60 km buffer displayed (n = 67 counties)

We focused on two of the four 2004 hurricanes which made landfall in Florida: Charley (August 13, 2004) and Ivan (September 21, 2004) (Fig. 1). Charley was the first and strongest hurricane of the season, hitting many Florida counties with diverse population groups. In contrast, Ivan hit the Florida panhandle, where the population that is socioeconomically and socially distinct from the rest of the state. Therefore, analyses comparing counties exposed to Hurricane Ivan to the rest of the state are uniquely susceptible to bias from unmeasured confounding. Therefore, analysis of Hurricane Ivan presents a opportunity to explore whether the differences-in-differences model accounts for unmeasured confounding that may bias analyses using general linear models.

Counties were classified with respect to hurricane exposure using two previously published methods. The first method applied exposure based on a cutoff of hurricane maximum wind severity according to the Saffir-Simpson Wind Scale [22]. Based on the cutoff for category 1 hurricane, counties affected by winds ≥74 mph were considered exposed and compared to counties with wind speeds less than 74 mph (unexposed). The second method was defined by a 60 km symmetrical spatial buffer around the storm track. Any county within the 60 km buffer, including partial counties, was considered exposed and compared to the counties completely outside of the buffer (unexposed) [23, 24]. We compared the two methods of classifying exposure to demonstrate the consistency of the results.

### Statistical methods

We calculated county-level live birth rates for 2003 and 2004 conceptions as the number of live births in a county divided by the total county population at midyear times 1000. All analyses were conducted in SAS 9.2 (Cary, North Carolina) and an example SAS program of difference-in-differences methods is provided in the Additional file 1: supplemental digital content 1. This research was approved by the Institutional Review Boards at the Florida Department of Health (#H13049) and the University of North Carolina at Chapel Hill (#13-0784).

#### Difference-in-differences

**Fig. 2:**
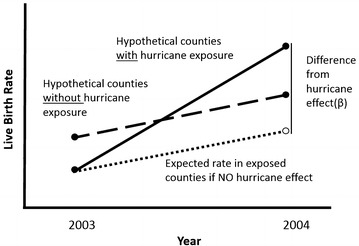
Hypothetical Illustration of difference-in-differences method

Difference-in-differences is a statistical technique which attempts to mimic experimental research study design for analyses of observational data. The effect of exposure (treatment) on an outcome is calculated as the difference of the average change in the exposed group minus the change in the unexposed group. In this hurricane exposure example, we are estimating the difference in live birth rate differences in exposed counties from the 2003 to 2004 time periods as shown in the hypothetical diagram Fig. 2. The change in birth rate labeled “Difference from hurricane effect” (graphically illustrates the rate estimated using the difference-in-differences analysis.

Difference-in-differences models have the same assumptions as the underlying model form (in this case general linear model) with additional assumptions regarding parallel trends in county attributes. This implies that within-county characteristics, e.g., median income, are invariant between time periods or change at the same rate across exposed and unexposed counties. If this assumption holds, then difference-in-differences removes confounding by these covariates, even those which are unmeasured. When this assumption is violated, there will be residual confounding by factors that change differentially between study years.

**Fig. 3:**
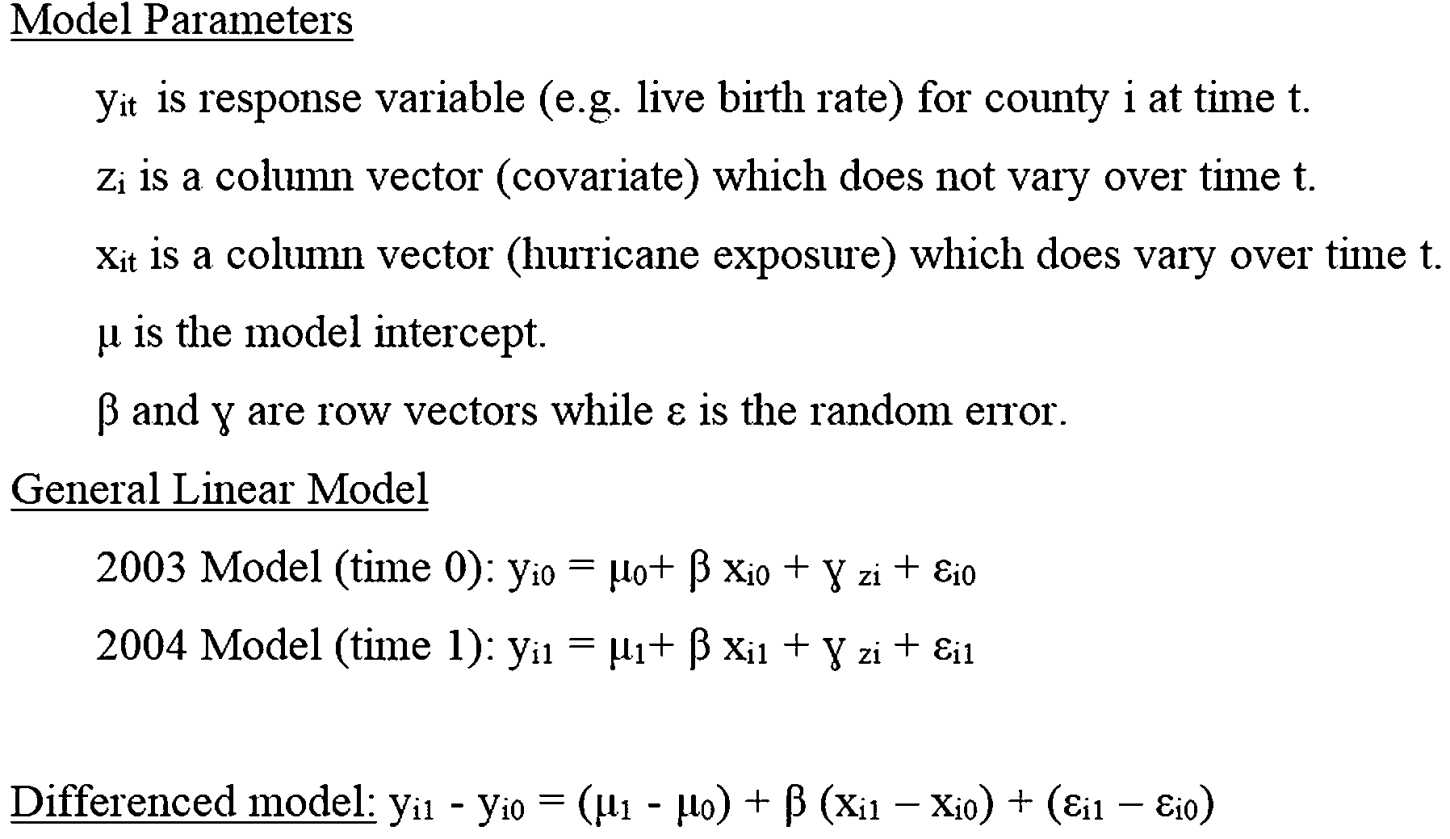
Methods description for general linear and difference-in-differences models

We conducted analyses of the difference-in-differences method using PROC GLM with the ABSORB statement in SAS 9.2 to estimate the rate difference between the 2003 and 2004 within-county live birth rates. This is analogous to the rate difference generated in a general linear model with the previous unexposed year rate difference removed, estimating the marginal within-county rate difference (Fig. 3). The 2003 conception period in these models stands in for the baseline differences in covariate distributions between counties before hurricane exposure or non-exposure in 2004.

#### General linear models

We illustrate sensitivity of results by fitting general linear models to estimate the association between hurricane exposure and county-specific live birth rates of 2004 conceptions. We conducted unadjusted and adjusted general linear models using PROC REG with identity link and Gaussian random distribution in SAS 9.2 software to estimate rate differences.

To demonstrate a regression approach to control confounding in aggregate analyses, the models were adjusted for county-level 2000 US Census covariates, including percent renter-occupied units, median household income, percent of persons who speak English less than well and percent of adults with more than high school education. These variables have been used previously in developing social indices and controls in county-level studies [25–27]. The chosen covariates were determined a priori based on a literature review of natural disasters and public health.

## Results

The first hurricane of 2004, Charley, moved northeast through central Florida impacting a large geographic area. In contrast, Hurricane Ivan made landfall in Alabama and Florida, affecting only a small area of the Florida panhandle. The Florida counties exposed to Hurricane Ivan had lower median incomes, less education and a higher proportion of renter occupied units than the unexposed counties (Table 2). The number of exposed and unexposed counties varied with the specific hurricane (Table 1). For example, using the 60 km buffer, twenty-three counties were classified as exposed to Hurricane Charley while only two were exposed to Hurricane Ivan.

**Table 1:** Census-adjusted general linear model (GLM) and difference-in-differences models of Hurricane exposure and live birth

Exposure method	Hurricane Charley exposure		Hurricane Ivan exposure	
	N exposed^a^	Estimate (95 % CI)	N exposed^a^	Estimate (95 % CI)
60 km buffer				
Within county difference-in-differences model	23	0.02 (−0.16 to 0.20)	2	−0.02 (−0.51 to 0.47)
Across County GLM adjusted model		−0.30 (−0.72 to 0.13)		2.80 (1.94 to 3.67)
Wind speed ≥ 74 mph				
Within county difference-in-differences model	5	0.18 (−0.13 to 0.49)	3	0.05 (−0.34 to 0.44)
Across county GLM adjusted model		0.06 (−0.67 to 0.78)		2.23 (1.47 to 2.99)

Rates per 1000 Individuals, Florida 2004 (n = 67 counties)

Adjusted models include percent renter-occupied units, median household income, percent of persons who do not speak English and percent of persons with more than high school education

*CI* confidence interval, *GLM* general linear model

^a^N exposed column indicates the number of exposed counties given indicated exposure method and hurricane out of 67 total counties

**Table 2:** Description of census county variables from adjusted analysis, Florida 2004 (n = 67 counties)

Census variable	Overall number counties	Charley exposed counties^a^ (n = 23)	Ivan exposed counties^a^ (n = 2)
	N (%)	N (%)	N (%)
Renter occupied units			
<15 %	5 (7.5 %)	1 (4.3 %)	0 (0.0 %)
15 to <25 %	43 (64.2 %)	13 (56.5 %)	1 (50.0 %)
25 to <35 %	12 (17.9 %)	7 (30.4 %)	1 (50.0 %)
35+ %	7 (10.4 %)	2 (8.7 %)	0 (0.0 %)
Median household			
<Median (38,819 K)	47 (70.1 %)	14 (60.8 %)	1 (50.0 %)
>Median (38,819 K)	20 (29.9 %)	9 (39.2 %)	1 (50.0 %)
Percent persons do not speak English			
<5 %	12 (17.9 %)	0 (0.0 %)	0 (0.0 %)
5 to <15 %	41 (61.1 %)	15 (65.2 %)	2 (100.0 %)
15 to <20 %	7 (10.4 %)	4 (17.4 %)	0 (0.0 %)
25+ %	7 (10.4 %)	4 (17.4 %)	0 (0.0 %)
Percent persons with ≥HS education			
<65 %	4 (6.0 %)	2 (8.7 %)	0 (0.0 %)
65 to <75 %	26 (38.8 %)	4 (17.4 %)	0 (0.0 %)
75 to <85 %	26 (38.8 %)	12 (52.2 %)	1 (50.0 %)
85+ %	11 (16.4 %)	5 (2.2 %)	1 (50.0 %)

^a^Exposure for Table 2 was categorized by 60 km buffer

Associations in Table 1 are reported as rate differences (RD) with 95 % confidence interval (95 % CI). For Hurricane Charley, neither the difference-in-differences nor the general linear models identified an association between hurricane and live birth rates. The 95 % confidence intervals produced by the difference-in-differences method exhibited greater statistical precision as shown by the tighter confidence intervals.

The associations found for Hurricane Ivan differed from Hurricane Charley. The difference-in-differences model did not suggest an association between hurricane exposure and live birth rates. In contrast, in the general linear models, live birth rates were consistently positively associated with both the 60 km buffer [RD = 2.80 births/1000 individuals (1.94, 3.67)] and the wind speed ≥74 mph [RD = 2.23 births/1000 individuals (1.47, 2.99)]. Higher birth rates in the exposed Panhandle counties, even after covariate-adjustment, are compatible with our hypothesis that GLM analysis of Hurricane Ivan would be subject to residual confounding from unmeasured social and socioeconomic factors that are more common in the Panhandle and associated with higher birth rates.

## Discussion

To assess the health impacts of environmental exposures including natural disasters, epidemiologists often conduct analyses of aggregated data. Such approaches may have methodological limitations, including incomplete confounder control, exposure misclassification and lack of group level covariate information. We sought to demonstrate an application of the difference-in-differences method in estimating the effect of county-level hurricane exposure on live birth rates. While still potentially suffering from bias due to residual confounding and migration, this method overcomes some of the limitations of conventional approaches by addressing confounding by unmeasured time-invariant attributes. It has become increasingly common in epidemiologic and public health research to perform aggregate level analysis (e.g., at the level of the county, ZIP code or census track) and to use aggregate indices or census variables to control for confounding (Platt et al. [26]). Results from the difference-in-differences analyses demonstrate a method to improve control of confounding due to unmeasured variables in aggregate analyses. We demonstrated the use of difference-in-difference into a spatial single year comparison; however, studies of other outcomes with hurricane exposure often use pre-post analysis or clinic-based populations. Methods like difference-in-difference modeling can be applied to control confounding in ecological pre-post or county-level analysis [28]. Overall across exposure metrics and hurricanes, the consistency of the difference-in-differences method suggests the integrity of this method over the general linear models.

There is no current consensus on the impact of hurricane exposure on reproductive health, with associations widely varying across studies [3, 29]. These mixed findings are potentially the result of varied mechanisms of exposure (e.g., stress, economical, injury etc.), variations in exposure definitions, dissimilar study populations, incomplete confounding control or potential heterogeneity in hurricane effects. Our analysis applied several exposure metrics over multiple hurricanes to examine some of these potential sources of inconsistencies. All difference-in-differences models yielded null associations for both hurricanes and for all exposure metrics. In contrast the general linear model yielded some potential associations with Hurricane Ivan. In supplemental analyses of the other hurricanes in the 2004 season, while some variability exists between the GLM and DID method, none are as stark a contrast as that seen in Hurricane Ivan. In analyses of Hurricane Frances and Jeanne, general linear models varied in magnitude and direction while we found consistently null associations using the difference-in-differences approach (Additional file 1: supplemental digital content 2). Additionally, the results from the Hurricane Ivan models are compatible with the hypothesis that uncontrolled confounding by unmeasured or imprecisely measured factors like low socioeconomic status, more widespread lack of medical access, and social norms around higher parity may bias GLM models of these data.

Suggested associations between hurricane exposure and live birth rates are largely anecdotal, including clinical observations and media reports. Two studies have focused on birth rates after hurricane occurrence. Cohan and Cole investigated live birth rates in twenty-four South Carolina counties in a time-series analysis before and after Hurricane Hugo and found that before the hurricane live birth rates decreased, while after the hurricane live birth rates increased [21]. Hamilton investigated county live birth rates in the Gulf Coast states (Louisiana, Mississippi and Alabama) following Hurricane Katrina and had mixed results depending on state [5]. While both studies controlled for measured population characteristics, the difference-in-differences approach (which additionally accounts for some unmeasured factors) may more fully adjust for differing population characteristics.

Difference-in-differences models assumptions regarding parallel trends can in theory be evaluated to the extent that all confounders’ distributions are available in both time periods. A review of the current literature suggests that the parallel trend assumption was likely met as hurricanes are thought of as an exogenous occurrence likely uncorrelated with changes in determinants of birth rates [30]. However, like many environmental studies, our exposure was defined by county of residence and therefore secular trends influencing birth rate may differ by county. Researchers should consider these assumptions in the application of the difference-in-differences method.

While the difference-in-differences model may have improved control of unmeasured confounder bias, other biases may persist. For instance, several articles have criticized the use of difference-in-differences methods using large datasets, where inappropriately small standard errors can incorrectly indicate significant relationships [28, 31]. This is a particular problem if outcomes are non-independent between subjects, which is not expected to be the case in our birth rate outcome. Our difference-in-differences models showed tighter 95 % confidence intervals than the general linear models, indicating smaller standard errors, however, we assume individual changes in conception are independent.

There were also limitations with our study methods. A major limitation of our study is that the four hurricanes hit Florida in 2004 in rapid succession, limiting our ability to understand independent hurricane effects. In particular, the Ivan-unexposed counties were affected by exposure to other hurricanes, which could bias our estimates towards the null. However, supplemental analysis did not indicate that additional hurricanes conferred additional risk on affected counties. Another limitation is that the number of counties exposed changed by the method of exposure categorization, thus rendering comparisons across method or hurricane difficult, especially using general linear models (Table 1). The county-fixed effects which are used in the difference-in-differences approach better allows for comparisons across models. Moreover, our reliance on Vital Statistics data prevents us from understanding the impact of early pregnancy loss as well as being unable to adjust for information on migration into or out of our study population.

Changes in live birth rates can be influenced by increases in the number of conceptions, migration into or out of the study area, and changes in fetal loss rates. While we are assuming migration into and out of our Florida cohort is equal, we have no way to document births that occurred outside of the state of Florida due to relocation or evacuation. Studies of the 2004 hurricane season estimate that between one-quarter and one-third of Florida’s population evacuated their homes prior to at least one hurricane; and many were evacuated several times [32].We assume the relocation of potentially exposed individuals could bias associations toward the null since people returning to exposed counties would have actually received no direct exposure by evacuating heavily influenced areas. Maternal exposure was defined based on residence at the time of delivery as listed on the birth certificate; however, we acknowledge that residence in a county throughout pregnancy has not been verified.

## Conclusion

In summary, we illustrate a method of inference for aggregate analyses to partially account for unmeasured confounding. Our analysis differs from much of the current epidemiological application of differences-in-differences method by demonstrating its application with county-level data. The inconsistency of the literature on hurricanes and reproductive health may be in part due to biases inherent in pre-post or regression-based county-level comparisons. Because of limited information on covariates in administrative data sources like those analyzed here, the DID method may be particular useful as the exploitation of aggregate-level “big data” increases. This example can aid future researchers in applying these methods to future studies.

## Additional file

10.1186/s12982-015-0042-7 **Supplemental Digital Content 1.** Model form and code for difference-in-differences method application. **Supplemental Digital Content 2.** Table of Florida 2004 unadjusted, census adjusted and difference-in-difference analysis of Hurricane exposure and live birth rates (n = 67 counties).

## Abbreviations

95 % CI: 95 % confidence intervals
DID: difference-in-diffrences
GLM: general linear model
km: kilometers
Mph: miles per hour
RD: rate differences
