# Supplementary material for: County-level hurricane exposure and birth rates: application of difference-in-differences analysis for confounding control
Source: Emerg Themes Epidemiol. 2015 Dec 22;12:19. doi: 10.1186/s12982-015-0042-7 (PMC4688997; doi:10.1186/s12982-015-0042-7)
Supplement: Supplementary file 1 — 10.1186/s12982-015-0042-7 Supplemental Digital Content 1. Model form and code for difference-in-differences method application. Supplemental Digital Content 2. Table of Florida 2004 unadjusted, census adjusted and difference-in-difference analysis of Hurricane exposure and live birth rates (n = 67 counties). [file 12982_2015_42_MOESM1_ESM.docx]

**Online Appendix: Supplemental Digital Content**

**Supplemental Digital Content 1.** Model Form and Code for Difference-in-Differences Method Application

Variables

y_it_ is response variable (e.g., live birth rate) for county i at time t.

z_i_ is a column vector (covariate) which does not vary over time t.

x_it_ is a column vector (hurricane exposure) which does vary over time t.

μ is the model intercept.

β and ɣ are row vectors while ε is the random error.

Differenced model: y_i1_ - y_i0_ = (μ_1_ - μ_0_) + β (x_i1_ – x_i0_)+ (ε_i1_ – ε_i0_)

**SAS**

Starting point model each year separately:

PROC REG DATA=data;

MODEL outcome0=exposure0; /* unexposed or timepoint 0*/

MODEL outcome 1 = exposure 1; /* unexposed or timepoint 1*/

RUN;

Manual differencing technique:

DATA diff;

SET data;

outcomediff=outcome1-outcome0;

exposurediff=exposure1-exposure0;

PROC REG DATA=diff;

MODEL outcomediff=exposurediff;

RUN;

Alternatively if you have repeated measures for county at time 0 and time 1 this will generate the same point estimates (but potentially not standard error);

PROC GLM DATA=data;

ABSORB county; /*ABSORB tells SAS not to generate the coefficient of change for each county but still uses said coefficients in the model*/

MODEL outcome= exposure time;

RUN;

**STATA**

ssc install diff

diff outcome_var [if] [in] [weight],

Required: period (varname) treated (varname) Optional: cov(varlist)

| **Supplemental Digital Content 2.** Table of Florida 2004 Unadjusted, Census Adjusted^a^ and Difference in Difference Analysis of Hurricane Exposure and Live Birth Rates ( n=67 counties) | | | | | | | | | | | | | | | |
| --- | --- | --- | --- | --- | --- | --- | --- | --- | --- | --- | --- | --- | --- | --- | --- |
|  | ***Any Hurricane Exposure*** | | | ***Hurricane Charley Exposure*** | | | ***Hurricane Frances Exposure*** | | | ***Hurricane Ivan Exposure*** | | | ***Hurricane Jeanne Exposure*** | | |
| **Exposure Method** | Estimate (95% CI) | | | Estimate (95% CI) | | | Estimate (95% CI) | | | Estimate (95% CI) | | | Estimate (95% CI) | | |
| 60 km buffer |  |  |  |  |  |  |  |  |  |  |  |  |  |  |  |
| Within County Difference-in-Differences Model | 0.002 | (-0.08 | 0.08) | 0.02 | (-0.16 | 0.20) | -0.04 | (-0.22 | 0.14) | -0.02 | (-0.51 | 0.47) | 0.03 | (-0.15 | 0.21) |
| Across County GLM Unadjusted Model | -0.05 | (-0.23 | 0.13) | -0.39 | (-0.80 | 0.02) | -0.06 | (-0.45 | 0.33) | 2.62 | (1.98 | 3.60) | 0.03 | (-0.38 | 0.44) |
| Across County GLM Adjusted Model | -0.02 | (-2.08 | 2.05) | -0.30 | (-0.72 | 0.13) | -0.52 | (-2.67 | 1.61) | 2.80 | (1.94 | 3.67) | 0.09 | (-0.31 | 0.49) |
| Wind Speed ≥ 74 mph (binary) |  |  |  |  |  |  |  |  |  |  |  |  |  |  |  |
| Within County Difference-in-Differences Model | -0.05 | (-0.25 | 0.15) | 0.18 | (-0.13 | 0.49) | -0.10 | (-0.37 | 0.17) | 0.05 | (-0.34 | 0.44) | -0.18 | (-0.40 | 0.04) |
| Across County GLM Unadjusted Model | 0.28 | (-0.17 | 0.73) | 0.04 | (-0.72 | 0.80) | -0.11 | (-0.76 | 0.54) | 2.01 | (1.83 | 2.19) | -0.17 | (-0.70 | 0.36) |
| Across County GLM Adjusted Model | 0.34 | (-0.78 | 0.88) | 0.06 | (-0.67 | 0.78) | -0.02 | (-0.48 | 0.43) | 2.23 | (1.47 | 2.99) | -0.17 | (-0.69 | 0.35) |
| Abbreviation: CI, confidence interval; GLM, general linear model  ^a^ Adjusted models include percent renter-occupied units, median household income, percent of persons who do not speak English and percent of persons with more than high school education. | | | | | | | | | | | | | | | |
